# 1-(2,6-Dihydr­oxy-4-methoxy­phen­yl)-3-phenyl­propan-1-one^1^

**DOI:** 10.1107/S1600536810013590

**Published:** 2010-04-21

**Authors:** Suchada Chantrapromma, Jutatip Jeerapong, Thongchai Kruahong, Surat Laphookhieo, Hoong-Kun Fun

**Affiliations:** a Crystal Materials Research Unit, Department of Chemistry, Faculty of Science, Prince of Songkla University, Hat-Yai, Songkhla 90112, Thailand; b Department of Chemistry and Center of Excellence for Innovation in Chemistry, Faculty of Science and Technology, Suratthani Rajabhat University, Mueang, Surat Thani 84100, Thailand; c Natural Products Research Laboratory, School of Science, Mae Fah Luang University, Muang, Chiang Rai 57100, Thailand; d X-ray Crystallography Unit, School of Physics, Universiti Sains Malaysia, 11800 USM, Penang, Malaysia

## Abstract

The title compound, C_16_H_16_O_4_, a dihydro­chalcone, was isolated from the rhizomes of *Etlingera littoralis*. The mol­ecule is twisted with a dihedral angle of 71.69 (6)° between the two aromatic rings. The propanone unit makes dihedral angles of 4.07 (6) and 73.56 (7)°, respectively, with the 2,6-dihydroxy-4-methoxyphenyl and phenyl rings. The meth­oxy group is approximately coplanar with the attached benzene ring with a dihedral angle of 1.74 (10)°. An intra­molecular O—H⋯O hydrogen bond generates an *S*(6) ring motif. In the crystal, inter­molecular O—H⋯O hydrogen bonds link the mol­ecules into chains along [201]. A π–π inter­action with a centroid–centroid distance of 3.5185 (6) Å is also observed.

## Related literature

For details of hydrogen-bond motifs, see: Bernstein *et al.* (1995 ▶). For bond-length data, see: Allen *et al.* (1987 ▶). For background to dihydro­chalcones and their activities, see: Nilsson (1961 ▶); Nowakowska (2007 ▶); Portet *et al.* (2007 ▶). For Zingiberaceae plants, see: Chuakul & Boonpleng (2003 ▶); Reanmongkol *et al.* (2006 ▶); Sirirugsa (1999 ▶); Tewtrakul, Subhadhirasakul & Kummee (2003 ▶); Tewtrakul, Subhadhirasakul, Puripattanavong & Panphadung (2003 ▶). For a related structure, see: Ng *et al.* (2005 ▶). For the stability of the temperature controller used in the data collection, see: Cosier & Glazer (1986 ▶).
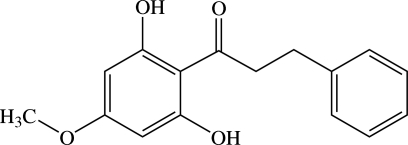


## Experimental

### 

#### Crystal data

C_16_H_16_O_4_
*M* *_r_* = 272.29
Monoclinic, 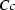

*a* = 7.2142 (6) Å
*b* = 30.522 (2) Å
*c* = 6.5587 (5) Å
β = 107.267 (2)°
*V* = 1379.09 (18) Å^3^
*Z* = 4
Mo *K*α radiation
μ = 0.09 mm^−1^
*T* = 100 K
0.46 × 0.34 × 0.18 mm

#### Data collection

Bruker APEX DUO CCD area-detector diffractometer
Absorption correction: multi-scan (*SADABS*; Bruker, 2009 ▶) *T*_min_ = 0.958, *T*_max_ = 0.983
17744 measured reflections
3044 independent reflections
2940 reflections with *I* > 2σ(*I*)
*R*_int_ = 0.025

#### Refinement

*R*[*F* ^2^ > 2σ(*F* ^2^)] = 0.034
*wR*(*F* ^2^) = 0.097
*S* = 1.08
3044 reflections
186 parameters
2 restraints
H atoms treated by a mixture of independent and constrained refinement
Δρ_max_ = 0.34 e Å^−3^
Δρ_min_ = −0.40 e Å^−3^

### 

Data collection: *APEX2* (Bruker, 2009 ▶); cell refinement: *SAINT* (Bruker, 2009 ▶); data reduction: *SAINT*; program(s) used to solve structure: *SHELXTL* (Sheldrick, 2008 ▶); program(s) used to refine structure: *SHELXTL*; molecular graphics: *SHELXTL*; software used to prepare material for publication: *SHELXTL* and *PLATON* (Spek, 2009 ▶).

## Supplementary Material

Crystal structure: contains datablocks global, I. DOI: 10.1107/S1600536810013590/is2537sup1.cif

Structure factors: contains datablocks I. DOI: 10.1107/S1600536810013590/is2537Isup2.hkl

Additional supplementary materials:  crystallographic information; 3D view; checkCIF report

## Figures and Tables

**Table 1:** Hydrogen-bond geometry (Å, °)

*D*—H⋯*A*	*D*—H	H⋯*A*	*D*⋯*A*	*D*—H⋯*A*
O2—H1*O*2⋯O1	0.82	1.71	2.4576 (11)	150
O4—H1*O*4⋯O2^i^	0.80 (3)	1.90 (3)	2.6920 (10)	175 (3)

Symmetry code: (i) 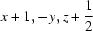.

**Fig. 1.**
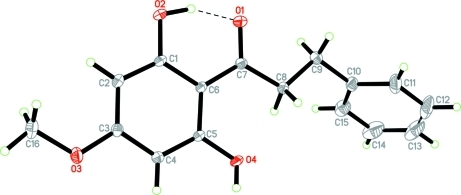
The molecular structure of the title compound, with 50% probability displacement ellipsoids and the atom-numbering scheme.

**Fig. 2.**
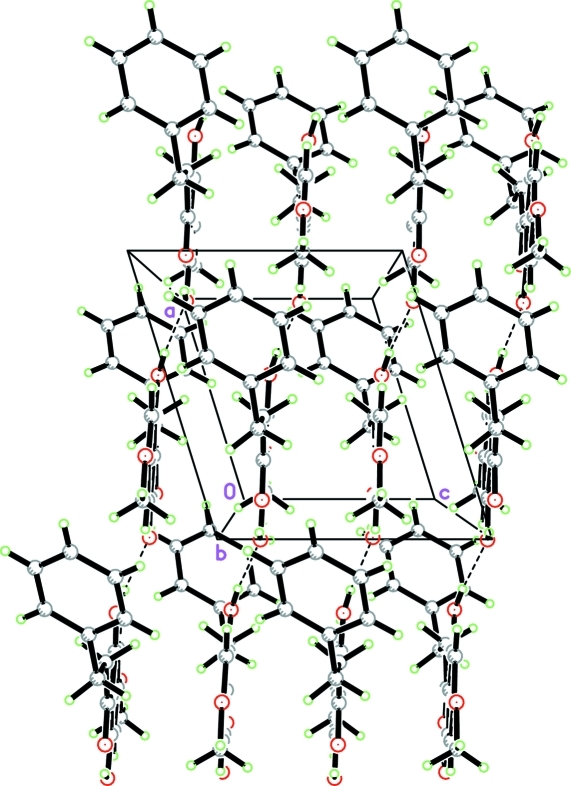
The crystal packing of the title compound viewed down the b axis, showing chains running along the [201] direction. Hydrogen bonds are shown as dashed lines.

## supplementary crystallographic information

### Comment

Zingiberaceae plants are the ground plants of tropical forests. Many of them are used for food, spices, medicines, dyes, perfume and aesthetics (Sirirugsa, 1999). Secondary metabolites from Zingiberaceae plants have found to be anti-inflammatory (Reanmongkol *et al.*, 2006), HIV-1 protease inhibitory (Tewtrakul, Subhadhirasakul & Kummee, 2003; Tewtrakul, Subhadhirasakul, Puripattanavong & Panphadung, 2003). *Etlingera littoralis* is one of the Zingiberaceae plants and its decoction of the rhizomes has been used for the treatment of stomachache, carminative and heart tonic (Chuakul & Boonpleng, 2003). As part of our study of chemical constituents and bioactive compounds from the rhizomes of *Etlingera littoralis* which were collected from Surat Thani province in the southern of Thailand, the title dihydrochalcone, (I), was isolated. Herein we report its crystal structure. The title compound was found to possess antibacterial (Nowakowska, 2007) and antiplasmodial activities (Portet *et al.*, 2007).

The molecule of the title dihydrochalcone (Fig. 1), C_16_H_16_O_4_, is twisted as the dihedral angle between the 2,6-dihydroxy-4-methoxyphenyl and phenyl rings is 71.69 (6)°. Whereas the 1-propanone unit (C7–C9/O1) makes the dihedral angles of 4.07 (6) and 73.56 (7)° with the C1–C6 benzene and C10–C15 phenyl rings, respectively. The two hydroxy and a methoxy groups are co-planar with the attached benzene ring with the *r.m.s.* of 0.0078 (1) Å for the ten non H atoms and the torsion angle C16–O3–C3–C2 = -1.66 (15)°. An intramolecular O2—H1O2···O1 hydrogen bond generates an S(6) ring motif (Bernstein *et al.*, 1995) (Fig. 1 and Table 1). The bond distances are of normal values (Allen *et al.*, 1987) and are comparable with the closely related structure (Ng *et al.*, 2005).

In the crystal packing (Fig. 2) , O—H···O hydrogen bonds (Table 1) formed between the two hydroxy groups link the molecules into chains along the [201] direction in which the adjacent chains are in anti-parallel manner. A π–π interaction with *Cg*1···*Cg*1 distance of 3.5185 (6) Å was observed (symmetry code x, -y, -1/2+z); *Cg*1 is the centroid of the C1–C6 benzene ring.

### Experimental

The fresh rhizomes of *E. littoralis* (3.89 kg) were chopped and extracted with 50% CH_2_Cl_2_-MeOH, over the period of 3 days at room temperature. The extraction was filtered and evaporated to dryness under reduced pressure to give crude extract which was further partitioned with water and CH_2_Cl_2_ to afford the dichloromethane extract (22.88 g). The portion of dichloromethane extract (11.80 g) was subjected to quick column chromatography (QCC) on silica gel eluting with a gradient of EtOAc–hexane to give thirteen fractions. Fraction F8 (322.4 mg) was washed with 20% CH_2_Cl_2_-hexane yielding solid which was further separated by column chromatography on silica gel with 70% CH_2_Cl_2_-hexane to give compound (I) (50.1 mg). Yellow block-shaped single crystals of the compound (I) suitable for *X*-ray structure determination were obtained from ethyl acetate by slow evaporation at room temperature after a few days, Mp 443 K. The NMR spectral data were consistent with the *X*-ray structure.

### Refinement

Hydroxy H atoms attached to O4 was located from a difference map and isotropically refined. The remaining H atoms were placed in calculated positions, with *d*(O—H) = 0.82 Å and *d*(C—H) = 0.93 Å for aromatic, 0.97 for CH_2_ and 0.96 Å for CH_3_ atoms. The *U*_iso_(H) values were constrained to be 1.5*U*_eq_ of the carrier atom for hydroxy and methyl H atoms and 1.2*U*_eq_ for the remaining H atoms. A rotating group model was used for the methyl groups. The highest residual electron densitypeak is located at 0.69 Å from C1 and the deepest hole is located at 0.84 Å from C7. A total of 2607 Friedel pairs were merged before final refinement as there is no large anomalous dispersion for the determination of the absolute configuration.

### Crystal data

**Table:**

C_16_H_16_O_4_	*F*(000) = 576
*M**_r_* = 272.29	*D*_x_ = 1.311 Mg m^−^^3^
Monoclinic, *C**c*	Melting point: 443 K
Hall symbol: C -2yc	Mo *K*α radiation, λ = 0.71073 Å
*a* = 7.2142 (6) Å	Cell parameters from 3044 reflections
*b* = 30.522 (2) Å	θ = 2.7–35.0°
*c* = 6.5587 (5) Å	µ = 0.09 mm^−^^1^
β = 107.267 (2)°	*T* = 100 K
*V* = 1379.09 (18) Å^3^	Block, yellow
*Z* = 4	0.46 × 0.34 × 0.18 mm

### Data collection

**Table:**

Bruker APEX DUO CCD area-detector diffractometer	3044 independent reflections
Radiation source: sealed tube	2940 reflections with *I* > 2σ(*I*)
graphite	*R*_int_ = 0.025
φ and ω scans	θ_max_ = 35.0°, θ_min_ = 2.7°
Absorption correction: multi-scan (*SADABS*; Bruker, 2009)	*h* = −11→11
*T*_min_ = 0.958, *T*_max_ = 0.983	*k* = −49→48
17744 measured reflections	*l* = −10→10

### Refinement

**Table:**

Refinement on *F*^2^	Primary atom site location: structure-invariant direct methods
Least-squares matrix: full	Secondary atom site location: difference Fourier map
*R*[*F*^2^ > 2σ(*F*^2^)] = 0.034	Hydrogen site location: inferred from neighbouring sites
*wR*(*F*^2^) = 0.097	H atoms treated by a mixture of independent and constrained refinement
*S* = 1.08	*w* = 1/[σ^2^(*F*_o_^2^) + (0.0648*P*)^2^ + 0.1812*P*] where *P* = (*F*_o_^2^ + 2*F*_c_^2^)/3
3044 reflections	(Δ/σ)_max_ = 0.001
186 parameters	Δρ_max_ = 0.34 e Å^−^^3^
2 restraints	Δρ_min_ = −0.40 e Å^−^^3^

### Special details

**Table:**

Experimental. The crystal was placed in the cold stream of an Oxford Cryosystems Cobra open-flow nitrogen cryostat (Cosier & Glazer, 1986) operating at 100.0 (1) K.
Geometry. All esds (except the esd in the dihedral angle between two l.s. planes) are estimated using the full covariance matrix. The cell esds are taken into account individually in the estimation of esds in distances, angles and torsion angles; correlations between esds in cell parameters are only used when they are defined by crystal symmetry. An approximate (isotropic) treatment of cell esds is used for estimating esds involving l.s. planes.
Refinement. Refinement of F^2^ against ALL reflections. The weighted R-factor wR and goodness of fit S are based on F^2^, conventional R-factors R are based on F, with F set to zero for negative F^2^. The threshold expression of F^2^ > 2sigma(F^2^) is used only for calculating R-factors(gt) etc. and is not relevant to the choice of reflections for refinement. R-factors based on F^2^ are statistically about twice as large as those based on F, and R- factors based on ALL data will be even larger.

### Fractional atomic coordinates and isotropic or equivalent isotropic displacement parameters (Å^2^)

**Table:**

	*x*	*y*	*z*	*U*_iso_*/*U*_eq_	
O1	0.57929 (10)	0.09273 (3)	0.16110 (12)	0.01772 (14)	
O2	0.40256 (10)	0.02283 (2)	0.09183 (12)	0.01659 (14)	
H1O2	0.4212	0.0494	0.0997	0.025*	
O3	0.76008 (12)	−0.11076 (2)	0.22402 (13)	0.01827 (13)	
O4	1.09458 (10)	0.02313 (2)	0.35682 (12)	0.01540 (13)	
H1O4	1.186 (4)	0.0088 (8)	0.419 (4)	0.043 (6)*	
C1	0.57479 (11)	0.00170 (3)	0.15654 (13)	0.01230 (14)	
C2	0.56979 (13)	−0.04392 (3)	0.15433 (14)	0.01400 (15)	
H2A	0.4524	−0.0589	0.1102	0.017*	
C3	0.74533 (14)	−0.06642 (3)	0.21997 (15)	0.01307 (13)	
C4	0.92225 (13)	−0.04409 (3)	0.28721 (14)	0.01272 (14)	
H4A	1.0381	−0.0597	0.3298	0.015*	
C5	0.92521 (11)	0.00137 (3)	0.29051 (12)	0.01140 (13)	
C6	0.75073 (14)	0.02604 (3)	0.22318 (15)	0.01112 (13)	
C7	0.74075 (13)	0.07385 (3)	0.21838 (14)	0.01271 (13)	
C8	0.91965 (13)	0.10221 (3)	0.27702 (14)	0.01421 (14)	
H8A	1.0023	0.0936	0.1911	0.017*	
H8B	0.9911	0.0971	0.4255	0.017*	
C9	0.87479 (16)	0.15107 (3)	0.24454 (17)	0.01880 (16)	
H9A	0.7893	0.1559	0.1013	0.023*	
H9B	0.8081	0.1608	0.3448	0.023*	
C10	1.05757 (16)	0.17743 (3)	0.27659 (18)	0.01983 (18)	
C11	1.1302 (2)	0.20381 (4)	0.4562 (2)	0.0303 (2)	
H11A	1.0662	0.2049	0.5601	0.036*	
C12	1.2988 (3)	0.22868 (4)	0.4815 (3)	0.0440 (4)	
H12A	1.3455	0.2463	0.6013	0.053*	
C13	1.3961 (2)	0.22708 (5)	0.3287 (3)	0.0453 (4)	
H13A	1.5074	0.2438	0.3455	0.054*	
C14	1.3276 (2)	0.20066 (5)	0.1517 (3)	0.0396 (3)	
H14A	1.3938	0.1992	0.0499	0.048*	
C15	1.15876 (19)	0.17616 (4)	0.1255 (2)	0.0273 (2)	
H15A	1.1128	0.1587	0.0049	0.033*	
C16	0.58350 (17)	−0.13568 (3)	0.16199 (18)	0.0230 (2)	
H16A	0.6136	−0.1664	0.1775	0.034*	
H16B	0.5142	−0.1294	0.0157	0.034*	
H16C	0.5046	−0.1279	0.2513	0.034*	

### Atomic displacement parameters (Å^2^)

**Table:**

	*U*^11^	*U*^22^	*U*^33^	*U*^12^	*U*^13^	*U*^23^
O1	0.0124 (3)	0.0166 (3)	0.0227 (3)	0.0027 (2)	0.0030 (2)	0.0000 (3)
O2	0.0085 (3)	0.0182 (3)	0.0212 (3)	0.0004 (2)	0.0016 (2)	−0.0017 (2)
O3	0.0201 (3)	0.0120 (3)	0.0216 (3)	−0.0026 (2)	0.0044 (2)	−0.0005 (2)
O4	0.0072 (2)	0.0147 (3)	0.0221 (3)	−0.0008 (2)	0.0010 (2)	0.0007 (2)
C1	0.0087 (3)	0.0161 (3)	0.0118 (3)	−0.0003 (3)	0.0025 (2)	−0.0004 (3)
C2	0.0119 (3)	0.0157 (3)	0.0139 (3)	−0.0030 (3)	0.0030 (3)	−0.0011 (3)
C3	0.0144 (3)	0.0129 (3)	0.0116 (3)	−0.0014 (3)	0.0034 (2)	−0.0004 (3)
C4	0.0111 (3)	0.0132 (3)	0.0134 (3)	0.0002 (3)	0.0029 (2)	0.0002 (3)
C5	0.0089 (3)	0.0135 (3)	0.0114 (3)	−0.0005 (3)	0.0025 (2)	0.0002 (3)
C6	0.0087 (3)	0.0128 (3)	0.0114 (3)	−0.0007 (3)	0.0022 (2)	−0.0004 (3)
C7	0.0117 (3)	0.0137 (3)	0.0124 (3)	0.0003 (3)	0.0032 (2)	−0.0002 (3)
C8	0.0130 (3)	0.0125 (3)	0.0161 (3)	−0.0013 (3)	0.0028 (3)	−0.0007 (3)
C9	0.0179 (4)	0.0126 (3)	0.0242 (4)	0.0011 (3)	0.0036 (3)	−0.0009 (3)
C10	0.0203 (4)	0.0112 (3)	0.0232 (4)	−0.0016 (3)	−0.0010 (3)	0.0012 (3)
C11	0.0361 (6)	0.0168 (4)	0.0293 (5)	−0.0027 (4)	−0.0035 (4)	−0.0040 (4)
C12	0.0459 (8)	0.0190 (5)	0.0472 (8)	−0.0115 (5)	−0.0170 (6)	0.0002 (5)
C13	0.0297 (6)	0.0271 (6)	0.0637 (10)	−0.0136 (5)	−0.0098 (6)	0.0190 (6)
C14	0.0266 (5)	0.0360 (7)	0.0532 (8)	−0.0074 (5)	0.0073 (6)	0.0202 (6)
C15	0.0251 (5)	0.0238 (5)	0.0308 (5)	−0.0043 (4)	0.0052 (4)	0.0054 (4)
C16	0.0263 (5)	0.0178 (4)	0.0227 (4)	−0.0090 (4)	0.0040 (4)	−0.0014 (3)

### Geometric parameters (Å, °)

**Table:**

O1—C7	1.2531 (11)	C8—H8B	0.9700
O2—C1	1.3516 (11)	C9—C10	1.5052 (14)
O2—H1O2	0.8200	C9—H9A	0.9700
O3—C3	1.3574 (11)	C9—H9B	0.9700
O3—C16	1.4349 (13)	C10—C11	1.3943 (15)
O4—C5	1.3445 (10)	C10—C15	1.3951 (17)
O4—H1O4	0.79 (3)	C11—C12	1.401 (2)
C1—C2	1.3928 (13)	C11—H11A	0.9300
C1—C6	1.4230 (12)	C12—C13	1.384 (3)
C2—C3	1.3917 (14)	C12—H12A	0.9300
C2—H2A	0.9300	C13—C14	1.379 (3)
C3—C4	1.3978 (13)	C13—H13A	0.9300
C4—C5	1.3877 (12)	C14—C15	1.3956 (18)
C4—H4A	0.9300	C14—H14A	0.9300
C5—C6	1.4201 (12)	C15—H15A	0.9300
C6—C7	1.4607 (11)	C16—H16A	0.9600
C7—C8	1.5061 (13)	C16—H16B	0.9600
C8—C9	1.5276 (13)	C16—H16C	0.9600
C8—H8A	0.9700		
			
C1—O2—H1O2	109.5	C10—C9—C8	111.21 (8)
C3—O3—C16	117.72 (9)	C10—C9—H9A	109.4
C5—O4—H1O4	115.4 (18)	C8—C9—H9A	109.4
O2—C1—C2	117.08 (8)	C10—C9—H9B	109.4
O2—C1—C6	120.02 (8)	C8—C9—H9B	109.4
C2—C1—C6	122.90 (8)	H9A—C9—H9B	108.0
C3—C2—C1	118.14 (8)	C11—C10—C15	118.14 (11)
C3—C2—H2A	120.9	C11—C10—C9	121.36 (11)
C1—C2—H2A	120.9	C15—C10—C9	120.50 (9)
O3—C3—C2	123.84 (9)	C10—C11—C12	120.52 (15)
O3—C3—C4	114.90 (9)	C10—C11—H11A	119.7
C2—C3—C4	121.26 (7)	C12—C11—H11A	119.7
C5—C4—C3	120.05 (8)	C13—C12—C11	120.27 (14)
C5—C4—H4A	120.0	C13—C12—H12A	119.9
C3—C4—H4A	120.0	C11—C12—H12A	119.9
O4—C5—C4	120.48 (8)	C14—C13—C12	119.88 (13)
O4—C5—C6	118.37 (7)	C14—C13—H13A	120.1
C4—C5—C6	121.15 (8)	C12—C13—H13A	120.1
C5—C6—C1	116.50 (7)	C13—C14—C15	119.88 (16)
C5—C6—C7	124.74 (8)	C13—C14—H14A	120.1
C1—C6—C7	118.76 (8)	C15—C14—H14A	120.1
O1—C7—C6	120.09 (8)	C10—C15—C14	121.29 (13)
O1—C7—C8	117.52 (7)	C10—C15—H15A	119.4
C6—C7—C8	122.38 (8)	C14—C15—H15A	119.4
C7—C8—C9	113.30 (7)	O3—C16—H16A	109.5
C7—C8—H8A	108.9	O3—C16—H16B	109.5
C9—C8—H8A	108.9	H16A—C16—H16B	109.5
C7—C8—H8B	108.9	O3—C16—H16C	109.5
C9—C8—H8B	108.9	H16A—C16—H16C	109.5
H8A—C8—H8B	107.7	H16B—C16—H16C	109.5
			
O2—C1—C2—C3	179.91 (8)	C5—C6—C7—O1	−178.29 (8)
C6—C1—C2—C3	−0.16 (14)	C1—C6—C7—O1	1.64 (14)
C16—O3—C3—C2	−1.66 (15)	C5—C6—C7—C8	2.74 (15)
C16—O3—C3—C4	178.47 (8)	C1—C6—C7—C8	−177.33 (8)
C1—C2—C3—O3	−179.62 (8)	O1—C7—C8—C9	−3.30 (12)
C1—C2—C3—C4	0.24 (14)	C6—C7—C8—C9	175.70 (8)
O3—C3—C4—C5	−179.79 (8)	C7—C8—C9—C10	−172.32 (8)
C2—C3—C4—C5	0.34 (14)	C8—C9—C10—C11	−108.05 (11)
C3—C4—C5—O4	179.38 (7)	C8—C9—C10—C15	72.21 (12)
C3—C4—C5—C6	−1.01 (14)	C15—C10—C11—C12	0.83 (17)
O4—C5—C6—C1	−179.33 (7)	C9—C10—C11—C12	−178.92 (11)
C4—C5—C6—C1	1.05 (13)	C10—C11—C12—C13	−0.5 (2)
O4—C5—C6—C7	0.60 (14)	C11—C12—C13—C14	−0.4 (2)
C4—C5—C6—C7	−179.02 (8)	C12—C13—C14—C15	1.0 (2)
O2—C1—C6—C5	179.46 (8)	C11—C10—C15—C14	−0.27 (17)
C2—C1—C6—C5	−0.46 (14)	C9—C10—C15—C14	179.47 (11)
O2—C1—C6—C7	−0.47 (13)	C13—C14—C15—C10	−0.6 (2)
C2—C1—C6—C7	179.60 (8)		

### Hydrogen-bond geometry (Å, °)

**Table:**

*D*—H···*A*	*D*—H	H···*A*	*D*···*A*	*D*—H···*A*
O2—H1O2···O1	0.82	1.71	2.4576 (11)	150
O4—H1O4···O2^i^	0.80 (3)	1.90 (3)	2.6920 (10)	175 (3)

Symmetry codes:  (i) *x*+1, −*y*, *z*+1/2.
